# Scorpionism by *Hemiscorpius* spp. in Iran: a review

**DOI:** 10.1186/s40409-018-0145-z

**Published:** 2018-03-02

**Authors:** Rouhullah Dehghani, Fatemeh Kamiabi, Malihe Mohammadi

**Affiliations:** 1 Social Determinants of Health (SDH) Research Center and Department of Environment Health, Kashan University of Medical Sciences, Kashan, Iran; 2 Faculty of Health, Kerman University of Medical Sciences, Kerman, Iran

**Keywords:** *Hemiscorpius*, Scorpionism, Scorpion venom, Emergency, Epidemiology

## Abstract

Scorpions are distributed throughout Iran and the genus *Hemiscorpius* is particularly important in this region. *Hemiscorpius lepturus* is the most significant species within the genus in the country. Since scorpionism provoked by *Hemiscorpius* comprises a medical emergency, the present study is focused on this important issue. In order to perform the present work, a review of the medical and health-related literature was carried out in several databases. The current findings indicate that six species of *Hemiscorpius* are found in 15 states of Iran, mainly in the south and southwest. Deaths caused by stings were reported only for two species. The morphological characteristics and geographical distribution of *H. lepturus* in Iran, its venom and the toxic compounds, epidemiologic data and clinical manifestations of envenomation as well as treatment for affected people are herein reviewed and described. *H. lepturus* venom toxicity differs from other Iranian scorpions regarding duration and severity. Scorpionism is an important public health problem in Iran, especially in southwest and south regions and in urban areas. It is more prevalent in children and young people. *H. lepturus* venom is primarily a cytotoxic agent and has hemolytic, nephrotoxic and to some extent hepatotoxic activity. The use of polyvalent antivenom to prevent scorpion sting symptoms is recommended. A well-planned health education program might be useful in preventing scorpionism.

## Background

Venomous animals are spread throughout the globe and play an important role in the terms of ecological balance. Most species of these animals use their venom for predation or defense. Some animal venoms may be life threatening for humans whereas nontoxic animals may bite a human in defense situations [1, 2]. Some venomous animals inject their toxins into their prey through stingers or other apparatus, whereas others use mouth annexes such as fangs in snakes or chelicerae in spiders. In the latter situation, the victim may be affected by venom and by the animal’s oral flora [3–6]. Annually, 5 to 7 thousand people are bitten by snakes in Iran, and the average mortality due to snakebite is 7 to 12 people per year [7, 8]. Although snakes are usually more fearsome than other venomous animals, scorpions are more important than other species in Iran.

There is a rich fauna of venomous animals in Iran, especially concerning scorpions species [9–12]. Deaths attributable to scorpion stings, which occur in all regions of the country, are more numerous than those caused by other venomous animals [13–16]. Iranian scorpions generally belong to three families: Buthidae, Hemiscorpiidae and Scorpionidae. Scorpion stings have been reported in the country for centuries since there are ancient religious and historical texts indicating their presence [17–19].

About 50,000 cases of scorpion stings are recorded in Iran annually. Epidemiological studies have showed that scorpion stings comprise the major type of envenomation in the country, with most cases happening in the southwest [20]. Clinical and laboratory symptoms of scorpionism following *Hemiscorpius* stings are clearly different from those reported for other medically important scorpion species [21, 22]. This phenomenon is observed in countries such as Iran, Iraq, Pakistan, Saudi Arabia, Oman, Yemen and United Arab Emirates [23]. Considering the importance of scorpionism by *Hemiscorpius* in Iran, the present study was carried out to investigate various aspects of this phenomenon in Iran.

A review of the medical and health-related literature was carried out in several databases including: Medline, Web of Science, Cochrane Library Database, Scopus, Google Scholar, SID and Iran Medex. Search terms were: scorpion, medical care, Iran, *Hemiscorpius*, *Hemiscorpius lepturus*, scorpion sting, antivenom, venom, symptom, epidemiology, clinical study and distribution. The results included Persian and English language studies from 1978 to 2016. In addition, sources of medicine history of Iran before and after Islam were accessed in order to study the history of scorpionism by *Hemiscorpius*.

The search strategy and review process were performed according to the aims of the study, with emphasis on studies conducted in Iran, restricted to the morphological characteristics and geographical distribution of *H. lepturus* in the country, its venom and the toxic compounds, epidemiologic data and clinical manifestations of envenomation as well as treatment with antivenom. A total of 193 studies matched the search terms, and 71 of them were excluded after initial screening.

### Hemiscorpiidae family

The number of species, genera and families of scorpions in the world have changed substantially in a relatively short time. By the last two decades, nearly a thousand species have been recognized and described globally [24–27]. So far, 2231 species of scorpions divided into 208 genera and 20 families have been recognized. According to the latest revision of the classification of scorpions in Iran, three families of Buthidae (51 species), Scorpionidae (three species) and Hemiscorpiidae (five species) were reported from different parts of the country [20].The Buthidae family is the largest and most abundant scorpion family in the world. Moreover, members of this family are considered the most dangerous ones [28–33].

The Hemiscorpiidae family has undergone several changes and developments during the last two decades. Because of the similarity between members of this family and those of Scorpionidae, they used to be considered as belonging to the same family in the past. Currently, this family consists of one genus (*Hemiscorpius*) and 15 species. Up to this moment, six species of this genus have been described in Iran (Table 1) [30, 34–36].

**Table 1:** The described species of *Hemiscorpius* genus in Iran

Genus	Species
*Hemiscorpius*	*Hemiscorpius acanthocercus* (Monod et Lourenço, 2005)
	*Hemiscorpius enischnochela* (Monod et Lourenço, 2005)
	*Hemiscorpius gaillardi* (Vachon, 1974)
	*Hemiscorpius lepturus* (Peters, 1862)
	*Hemiscorpius persicus* (Birula, 1903)
	*Hemiscorpius kashkayi* (Karataş and Gharkheloo 2013)

*Hemiscorpius lepturus* is a dangerous species that has been found in Iran, Oman, Iraq, Saudi Arabia, Yemen, Pakistan and United Arab Emirates [37–40]. This scorpion has highly cytotoxic venom that can cause scorpionism in humans and other animals. Some researchers believe that scorpionism may be related to several species of *Hemiscorpius*, not only *H. lepturus*. Therefore, it is important to be able to recognize the involved species after an incident, specially because *Hemiscorpius* species are very similar morphologically [41]. There are records of deaths attributable to *H. lepturus* and *H. acanthocercus*.

### Geographic distribution of *Hemiscorpius* genus

**Fig. 1:**
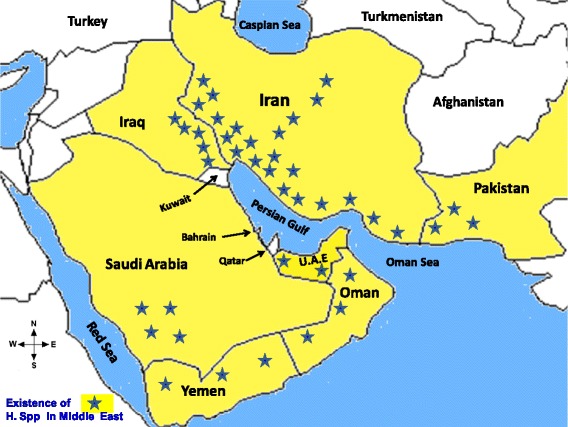
Distribution of *Hemiscorpius* species in Middle East (image prepared by R. Dehghani, reproduced with permission)

The Hemiscorpiidae family is spread throughout six countries in the Middle East, including Iran, Iraq, Pakistan, Saudi Arabia, Oman, Yemen and United Arab Emirates (Fig. 1) [23].

**Fig. 2:**
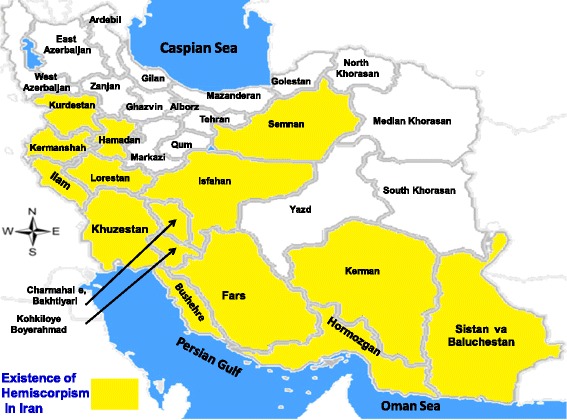
Distribution of *Hemiscorpius* species in Iran (image prepared by R. Dehghani, reproduced with permission)

These scorpions are usually find in hot and humid areas. In Iran, they have been reported in the provinces of Khuzestan, Semnan, Fars, Kurdistan, Hormozgan, Sistan and Baluchestan, Isfahan, Bushehr, Kohgiluyeh and Boyer-Ahmad, Ilam, Chahar Mahal and Bakhtiari, Lorestan, Hamedan, Kermanshah and Kerman (Fig. 2) [20, 40, 42–45].

### The historical background of scorpinism by *Hemiscorpius* in Iran

The history of *Hemiscorpius* stings in Iran dates back a long time [46, 47]. One of the first reports of evenomation by *Hemiscorpius* in Iran was presented by Avicenna in *The Canon of Medicine*, an encyclopedia of medicine in five books first published in the eleventh century. He described the effects of venomous animal bites on humans as well as possible treatments. In addition, measures to protect humans from envenomation by animals were described.

The report was based on documents of scorpionism syndrome patients from Khuzestan. Avicenna wrote: “As a result of Gadim [the local name of *Hemiscorpius* spp. in Khuzestan] sting, the person does not feel any pain immediately after it. One or two days later, the injured person experiences problems caused by the sting. After that, he or she becomes a very depressed person with a pale face that may be suffering from jaundice. Tongue is swelling; the sting location is festering; urine is red colored, which means it is bloody. The patient may be suffering from constipation. Heart rate increases and the person faints, which may lead to death. It should be noted that if there is less pain, a doctor should not frail or gullible, because Gadim is highly toxic and deceptive” [48, 49]. In addition to the clinical symptoms of *Hemiscorpius* stings, Avicenna also described the morphological characteristics and habitat of this scorpion species in Khuzestan and Iraq [48, 49].

The Academy of Gondishapur, also known as The Jondishapur University, was one of the three Sasanian centers of education and academy of learning in the city of Gundeshapur, Khuzestan Province, in Iran during late antiquity. It was considered the most important medical center of the ancient world during the 6th and 7th centuries. Since the capital of Iran during the Sassanian period was Ctesiphon or Mada’in, which was an area greatly affected by scorpionism by *Hemiscorpius*, numerous studies were carried out about this medical problem by researchers of the Academy of Gondishapur [50, 51].

The clinical manifestations of *Hemiscorpius* envenomation are quite infrequent, because its venom is one of the only presenting cytotoxic and hemolytic activities. Currently, the sting of *Hemiscorpius* species has been reported in several provinces of Iran, especially Khuzestan and Hormozgan, with significant clinical symptoms in victims, particularly children [52–56]. In traditional Iranian medicine, heating the sting site is one of the first measures. In the region of Rāmhormoz, people usually apply hot sand or hot stones immediately at the sting site [48, 49].

### The *Hemiscorpius* genus

**Fig. 3:**
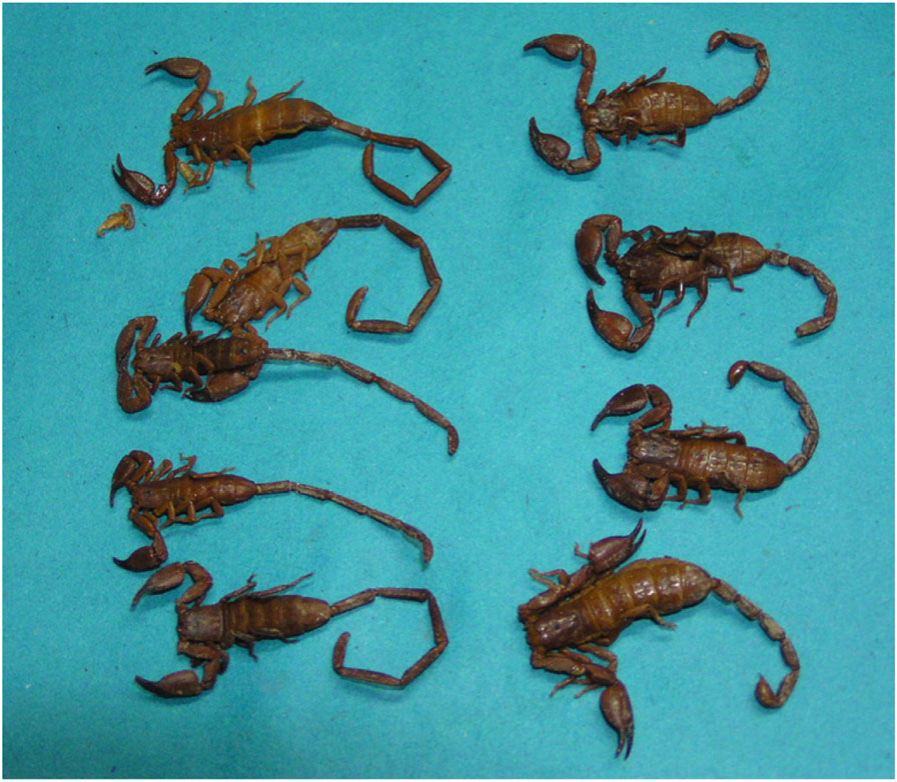
A photographic illustration of males (on the left) and females (on the right) specimens of *Hemiscorpius lepturus*. Note the very short stinger size and longer tail in males (copyright by R. Dehghani, reproduced with permission)

*Hemiscurpius* are non-digger scorpions usually the length of females and males reaches to 5 and 8 cm, respectively (males have a longer tail). Therefore, they present sexual dimorphism and their body color is transparent to turbid yellow. The pedipalps and legs are lighter in color, while moveable and fixed fingers of pedipalps are reddish brown. Brown spots can be observed at the end of the legs. The moving finger of chelicera have two branches (Fig. 3) [21, 22, 57].

*H. lepturus* venom is mainly composed of hemotoxins and cytotoxins [22]. A peptide isolated from *H. lepturus* venoms is hemicalcin, a calcium channel blocker that represents 0.6% of its crude venom. Intracerebroventricular injection of 300 ng of hemicalcin in mice induces neurotoxic symptoms in vivo, followed by death [58]. Hemitoxin is a potassium channel inhibitor that represents about 0.1% of crude venom [59]. Likewise, heminecrolysin, with dermonecrotic activity, and hemilipin (phospholipase A_2_) inhibited angiogenesis both in vitro and in vivo [60–62].

One of the most abundant components of *H. lepturus* venom are the enzymes that play important roles in scorpion venoms. Different enzymes including phospholipases, metalloproteases, hyaluronidases and proteases were identified by transcriptome analysis in *H. Lepturus* venom. The ester bond of phospholipids, the most important component of the cell membrane is hydrolyzed by phospholipases. Whole components of *H. lepturus* venom were identified by analyzing the venom gland transcriptome of this Iranian scorpion [63].

### Scorpionism by *Hemiscorpius*

#### Epidemiological data

Scorpion stings are a major public health problem in south and southwestern Iran. Although scorpion stinga are reported in all provinces of the country, most cases occur in the provinces of Khuzestan, Hormozgan, Sistan and Baluchestan, Bushehr and Ilam [20].

The average incidence of scorpionism was estimated to be 59.5 cases per 1 hundred thousand inhabitants and 19.5 deaths take place annually in Iran [20]. However, some studies indicate that the real number of annual stings is much higher than that one officially reported [64, 65]. Whereas the province of Mazandaran, in the northern region, has the lowest rate of cases (near zero), Khuzestan, in the southwest of Iran, has the highest one, reaching 541 cases per hundred thousand people [66].

Among the various species of scorpions in Iran, deaths are usually reported by stings of *Androctonus crassicauda*, *H. lepturus and H. acanthocercus*. The envenomation provoked by *H. lepturus* stings is significantly different from that one of the Buthidae family in severity and duration [21, 67]. The frequency of deaths caused by *H. lepturus* (Gadim) is higher [21, 67, 68].

Data collected during 5 years from two hospitals of the city of Ahvaz (center of Khuzestan) showed that *H. lepturus* was responsible for 10 to 15% of all hospital referred scorpion stings and 92% of all hospitalized victims of scorpion stings [21, 69].

Although scorpion stings were reported in southern and southwestern regions of Iran throughout the year, studies indicated that most stings happened in the warm months in spring and summer [15, 16, 21, 69–76]. A positive significant correlation between the frequency of scorpion stings and the environment temperature as well as the sunshine duration was reported [73]. However, the correlation between stings and relative humidity as a climate factor was negative [77].

Epidemiological analysis indicated that more than 50 to 60% of patients were from urban regions [15, 21, 69, 72, 76, 78]. Some studies conducted on age and sex distribution of scorpionism by *Hemiscorpius* indicated that most patients were female [71, 75, 78]. Other works, nevertheless, revealed that men were more at risk of scorpion stings than women [15, 66, 70, 72, 74]. Contrarily, a few studies reported that the frequency of recorded scorpion stings in both genders was nearly similar and there was not any significant difference between male and female in all age groups [16, 21, 69, 79]. Another observation was that women were more stung by scorpions in indoor places, whereas men were more at risk in the outdoors [77].

According to some findings, children were at greatest risk of scorpionism by *Hemiscorpius*. Envenomation is usually more severe in children and the mortality is higher among them [78, 80]. Research indicates that children under 10 years old and young people (aged between 10 and 20 years) were the first and second age groups, respectively, more at risk of scorpionism by *Hemiscorpius* in Iran [21, 69, 78, 81].

Most scorpion stings affect the lower part of the body (legs) [21, 71, 72]. However, other sting sites included the trunk, hands, head and neck [21]. Stings occurred mainly at night from 7 p.m. to 5 a.m. and in the early morning from 5 to 12 a.m. [21, 69].

The time interval observed between Gadim stings and antivenom treatment was less than 6 h for 56.6% of the cases, 6 to 12 h for 21% of cases, more than 12 h for 11% of cases and the rest of the victims did not received any antivenom [66]. A single study showed that the average time interval was less than 4 h for more than 70% of the hospitalized patients [21].

#### Clinical data

Scorpionism by *Hemiscorpius* may affect vital organs, with deadly consequences. The length of hospitalization of affected patients is greater than the that of patients stung by any other species in Iran. The delayed effects of the venom and its retention in the body are the main reasons for this phenomenon [81, 82].

##### Local symptoms

It has been reported that *H. lepturus* stings on the body are not easily recognized because of their small size (about 1 mm long). In comparison, black scorpion (*A. crassicauda*) stings that have about 6 to 8 mm are easily found [14, 81]. Since local pain due to *H. lepturus* stings is minimal, patients usually do not complain and do not seek medical help until the toxicity has already been established. The initial symptoms only include a minor itching at the sting site and mild pain. Therefore, local manifestations 24 h after the sting may be considered negligible. Buthidae stings, on the other hand, such as those by *Odonthobuthus doriae*, are more painful in the first moments. Symptoms such as local redness, inflammation, gangrene, necrosis, ecchymosis and blisters appears about 24 h after *H. lepturus* stings [83–85]. Consequently, some patients look for medical aid after a few days, when the sting site is inflamed and painful. Over time, the venom penetrates the dermis and adjacent tissues and causes cellulite and severe inflammation. An intense pain and cellulitis in the sting area may be seen. The most important consequences of *Hemiscorpius* stings, especially in children, are local necrosis, swelling, erythema and ecchymosis [21, 22, 69, 81, 86].

If the scorpion stinger passes though the epidermis and reaches capillaries and lymph system, then the effects of venom will be more severe. In some of these cases, venom may reach blood and lymph systems, causing non-uniform ecchymosis patches. The diameter of this non-uniform ecchymosis is about 25 cm, but sometimes does not lead to gangrenous cellulitis and sore. In these patients, systemic symptoms are rare and ecchymosis after one or 2 weeks, by itself without any action, will be improved [22, 87, 88]. Gadim scorpion venom caused skin sore or necrotic areas with 20–25 mm diameter in experimental rats. It was reported that any systemic problem was not found and animals remained alive [53, 89]. In children, stings on the hands and feet lead to severe swelling and inflammation due to the soft thin tissue of these areas. Stings on the neck, face and body in children and adult patients often are followed by more cellulite and swelling and they may be more dangerous in terms of sting site. After a while, the surface area of gangrene is cut out and sores appear through the fatty tissue under the skin [20, 22]. Extensive wounds only heal with skin grafts. It seems that in people or laboratory animals that have severe allergic reactions, the venom movement is stopped due to ulcers and necrosis. This means that the immune system with severe skin inflammation prevents blood and oxygen from reaching the sting site, except at the injection area [20, 88].

##### Systemic effects

Cardiotoxic effects of *H. leptorus* envenomation in humans have been reported. However, studies on the envenomation by this scorpion revealed that there are arrhythmogenic and negative inotropic effects in animal models [56]. Gadim venom has significant effects on white blood cells and, therefore, causes erythematous rashes and petechial hemorrhagic into the skin [90, 91]. Scorpion venoms, especially the one from Gadim, have also demonstrated hemolytic activity in vitro and it has been observed that the larger the amount of venom, the greater the extension of hemolytic damage [92, 93]. Patients stung by Gadim, especially children, can report these symptoms, but they are usually mild in adults and their effects may continue for 2 to 3 weeks without treatment [86, 94]. Among children, Gadim venom provokes a significant reduction in hematocrit rates [79].

Acute kidney injury (AKI) is frequently observed in children stung by *H. lepturus*. It was reported that for young patients, the delay in receiving medical care may result in pigmenturia, microangiopathic hemolysis, anemia, proteinuria and pyuria, which are predictors for AKI in children. Hemoglobinuria was found in more than 50% of *H. lepturus* sting cases. Hemolytic uremic syndrome after *H. lepturus* sting has also been reported [55, 79, 86, 95–99].

Hemoglobinuria, coagulopathy, transfusion and kidney problems were more observed in patients who had been admitted to the hospital after 24 h following the sting. Kidney problems were present only in patients with blood cell lysis and hemoglobinuria. The rates of hemoglobinuria were 93.8% in *H. lepturus* stung children [81]. Delayed hospital admission was recorded in cases of death. Therefore, clinical manifestations following *H. lepturus* envenomation including late renal failure are mainly time-dependent [24, 100]. Death due to *H. lepturus* envenomation may be provoked by cardiovascular or renal failure [83].

Overall, general signs such as seizures, distraction, irritability, restlessness, nausea, vomiting, headache, and possibly cyanosis have central nervous origin. These symptoms indicate the severity of envenomed patients. The central nervous system manifestations are seen mainly among children victims. Mental disorders as restlessness, distractibility, delusions and inappropriate behavior appears and usually return to normal as the patient improves. However, some patients, even after recovery, suffer from mental disorders such as increased aggressiveness, irritability, depression and emotional imbalance for longer periods [18, 21, 22, 69, 83].

It has been reported that the main clinical signs of *H. lepturus* envenomation are similar to those caused by spider bites of the genus *Loxosceles* (*L. reclusa* and *L. intermedia*). Loxoscelism is the only proven arachnological cause of dermonecrosis in the Western Hemisphere, particularly in the tropical urban regions of South America. There are many similarities in the toxic manifestations of the venom from this spider and the venom from *H. lepturus* in terms of the dermonecrosis, nephrotoxicity and direct hemolytic activities. Hemolysis causing loss of approximately 15% of the blood volume within 72 h [21, 62, 69, 81, 101–112].

#### Serotherapy with antivenom

Over the last 30 years, the most frequently used approach in the treatment of scorpionism in Iran is serotherapy with multivalent antivenom against six common Iranian scorpions [113]. The multivalent scorpion antivenom is presented as 5-mL ampoules of a pepsin digested refined and concentrated preparation, obtained from equine hyperimmune serum, stored at 2 to 8 °C. This antivenom is produced by Razi Institute of Vaccine and Serum Production in Karaj, Iran. It has specific potency against the venoms of the six endemic Iranian scorpions (*Androctonus crassicauda*, *Buthotus saulcyi*, *Buthotus schach*, *Odontobuthus doriae*, *Mesobuthus eupeus* and *H. lepturus*) [113].

Currently in Iran, stung patients, including those stung by *H. lepturus*, are treated in most clinical centers with intramuscular injection of available multivalent scorpion antivenom [93]. It has been reported that more than 95% of scorpion sting patients received the polyvalent antivenom. Despite the immediate administration of one or two doses of polyvalent antivenom intramuscularly at the hospital, in some severe cases, signs and symptoms have been observed. This may be due to the elapsed time between sting and treatment or improper dosage [93]. It is known that the efficacy of antivenom for reversal of cytotoxic manifestations following envenomation is time-limited. Therefore, using antivenom as early as possible before the occurrence of cytotoxic effects on different organs is recommended [114]. The time of antivenom injection has an important role in the effectiveness of antivenom. The epidemiological data and results of studies on experimental animals showed that the chance of preventing the occurrence and progress of systemic signs increases significantly when antivenom is administrated early. If any systemic signs or symptoms are observed in victims, it is recommended the intravenous administration of antivenom [115–117].

## Conclusion

The general results of this literature review indicate that the toxicity of *H. lepturus* stings differ from other Iranian scorpion stings in relation to duration and severity. Even the sting of this scorpion is without acute pain. Scorpionism by *Hemiscorpius* may related to several species, not only *H. lepturus*. Hence, recognizing the species involved in an envenomation case is more important. *Hemiscorpius* species are morphologically very close to each other. Misidentification may induce great mistakes in the final interpretation of results, which can only lead to more inefficacy in the treatment of disorders caused by dangerous scorpion species [41, 118].

Clinical symptoms and mortality from scorpion stings are related to two main factors: the characteristics of the stung patient (such as age and health condition) and the characteristics of the scorpion (such as species and venom potency) [77, 119, 120]. Scorpionism by *Hemiscorpius* is an important public health issue in Iran, especially in urban areas of the southwest and south regions. It is more prevalent in children and young people, whereas the mortality is greater in children. Most cases are recorded in the warm months of spring and summer at night. The dermal symptoms and systemic manifestations caused by *H. lepturus* indicates that venom of this species is mixture of toxins that is differs from the other Iranian scorpion species. *H. lepturus* venom is primarily a cytotoxic agent and has hemolytic, nephrotoxic and to some extent hepatotoxic activities.

The use of polyvalent antivenom to prevent different symptoms in scorpionism is recommended. According to evidence, the cardiovascular manifestations of stung patients, who received antivenom as soon as possible, were mild and the hemoglobinuria and renal failure were delayed as well [121, 122].

The high incidence of scorpionism by *Hemiscorpius* in Iran, especially in Khuzestan, suggests the necessity of preventive programs for decreasing the incidence of this phenomenon. One of the main challenges of controlling scorpionism is promotion of effective participation and awareness among the population. A well-planned health education program might be useful in preventing stings. Promoting health education, mainly among groups who are more at risk, about preventive measures against scorpions integrated with environmental measures and sanitary education of communities should be goal of control campaigns.
